# Association between polymorphism in *CDKN2B-AS1* gene and its interaction with smoking on the risk of lung cancer in a Chinese population

**DOI:** 10.1186/s40246-019-0240-4

**Published:** 2019-11-27

**Authors:** Xiaoting Lv, Zhigang Cui, Hang Li, Juan Li, Zitai Yang, Yanhong Bi, Min Gao, Ziwei Zhang, Shengli Wang, Baosen Zhou, Zhihua Yin

**Affiliations:** 1 Department of Epidemiology, School of Public Health, China Medical University, Shenyang, 110122 People’s Republic of China; 2 Key Laboratory of Cancer Etiology and Intervention, University of Liaoning Province, Shenyang, 110122 People’s Republic of China; 3 School of Nursing, China Medical University, Shenyang, 110122 People’s Republic of China

**Keywords:** Interaction, Lung cancer, lncRNA, Single nucleotide polymorphism

## Abstract

**Background:**

Long non-coding RNAs became the hot spots in the carcinogenesis of various tumors. This case-control study evaluated the association between the rs2151280 in lncRNA *CDKN2B-AS1* and lung cancer risk.

**Methods:**

This study included 507 lung cancer patients and 542 healthy individuals. Odds ratios and their 95% confidence intervals were calculated by unconditional logistic regression analysis to evaluate the association between the rs2151280 and lung cancer risk.

**Results:**

Compared with individuals carrying TT genotype, individuals carrying CC genotype of rs2151280 had a decreased risk of lung cancer (OR = 0.640, 95%CI = 0.421–0.972, *P* = 0.036). In the recessive model, rs2151280 CC genotype was observed to reduce the risk of lung cancer (OR = 0.684). C allele was associated with non-small cell lung cancer risk (OR = 0.674). The rs2151280 was significantly associated with lung adenocarcinoma risk (CCvsTT: OR = 0.567, 95%CI = 0.333–0.965, *P* = 0.037; CCvsTC+TT: OR = 0.543, 95%CI 0.330–0.893, *P* = 0.016, respectively). However, there was no significant association between rs2151280 and lung squamous cell carcinoma risk in five models. The quantitative analysis suggested that there were no significant interactions of rs2151280 with smoking exposure to lung cancer susceptibility.

**Conclusions:**

This hospital-based case-control study suggested that *CDKN2B-AS1* rs2151280 T>C was associated with the risk of lung cancer.

## Introduction

Lung cancer is a complex and malignant cancer with the high morbidity. Both environmental and genetic factors were acknowledged to the most important pathogenic factors for lung cancer. Lung cancer is divided into non-small cell lung cancer (NSCLC) and small cell lung cancer. NSCLC includes four major types: adenocarcinoma, squamous cell carcinoma, large cell carcinoma, and adenosquamous carcinoma. Age, lifestyle [1], genetic , endocrine [2], occupational exposure [3, 4], and other environmental factors [5] play important roles in lung cancer. In the past years, studies had shown that smoking was the strongest environmental risk factor for the occurrence and development of lung cancer. However, it was also reported that 25% of lung cancer patients were non-smokers. It suggested that genetic risk factor might play an important role in lung cancer. At the same time, some molecular epidemiological studies had suggested that the interaction between genetic and environmental factors might affect the incidence and development of lung cancer. However, the exact etiology and pathogenesis of lung cancer remained obscure.

With the in-depth study of high-throughput technologies, many genome-wide association studies (GWASs) had found the correlation between long non-coding RNAs (lncRNAs) and multiple cancers. LncRNAs, a class of non-coding RNA, ranging from 200 to 100 kbp in length, included non-protein coding transcripts [6]. While the lncRNAs were regarded as the “transcribed noise” in the early years, increasing evidence had shown that lncRNAs played key roles in biological processes. As regulators in cellular processes, lncRNAs could inhibit cell growth [7] and influence cell differentiation [8] and cell metastasis [9]. LncRNAs were known to play critical roles in multiple cancers by regulating multiple molecular regulation [10]. *CDKN2B-AS1* is an lncRNA (3.8 kb) transcribed from the *INK4B-ARF-INK4A* gene, locating on human chromosome 9p21 [11, 12]. GWAS had shown that SNPs in this region (9p21) were associated with many diseases, including cancers [13, 14]. Many studies had shown that the oncogenic properties of *CDKN2B-AS1* was existed in multiple carcinomas (thyroid cancer [15], gastric cancer [16], lung cancer [17, 18], and so on). Compared with normal controls, the overexpression of *CDKN2B-AS1* was obviously found in NSCLC tissues and serum samples [19]. The expression level of *CDKN2B-AS1* was higher in human non-small cell lung cancer than normal diploid fetal lung fibroblasts. The proliferation of H1299 cells were regulated by lncRNA *CDKN2B-AS1* [20]. The c-Myc was a closely-watched transcription factor which was overexpressed in NSCLC [21]. The c-Myc could directly transactivate *CDKN2B-AS1*, and *CDKN2B-AS1* could promote the proliferation of NSCLC cells [22].

A meta-analysis of 16 GWASs identified a novel disease locus for squamous cell carcinoma at 9p21. The result also found that 9p21.3 variants in the region of *CDKN2B-AS1* were associated with squamous cell lung cancer [23]. So the functional genetic variations in the lncRNA *CDKN2B-AS1* might contribute to the susceptibility of lung cancer. rs2151280 was located within the lncRNA *CDKN2B-AS1* at 9p21.3. Because rs2151280 might affect the expression of *CDKN2B-AS1* [24], and *CDKN2B-AS1* expression might affect the susceptibility of lung cancer, we suspected that rs2151280 might affect the susceptibility of lung cancer. Emerging evidence demonstrated that rs2151280 was related to various kinds of malignant tumors, which indirectly confirmed the importance of rs2151280 in cancer susceptibility. rs2151280 was initially discovered by a GWAS research on the correlation between rs2151280 and the risk of basal cell carcinoma, and the results showed that rs2151280 was related to the risk of basal cell carcinoma (BCC) [25]. The high expression of *CDKN2B-AS1* and the low expression of p14ARF were associated with rs2151280 TT variant in peripheral blood mononuclear cells (PBMCs) [26]. Molecular epidemiological studies had shown that rs2151280 was associated with various cancer risks (BCC [25] and plexiform neurofibromas (PNF) [24]). The association between *CDKN2B-AS1* rs2151280 and esophageal squamous cell carcinoma (ESCC) was not found in the study [27]. The relationship between *CDKN2B-AS1* rs2151280 and the risk of lung cancer was still unclear in the world.

Because the association between *CDKN2B-AS1* rs2151280 and lung cancer was undefined, our research had some innovation in investigating the association between *CDKN2B-AS1* rs2151280 and lung cancer risk. Therefore, we decided to further explore this SNP by supporting the above evidence in 507 lung cancer patients and 542 cancer-free controls. And we also evaluated the interaction of rs2151280 with smoking status on the risk of lung cancer in the case-control study. As far as we know, this was the first time to evaluate the effect of rs2151280 in lncRNA *CDKN2B-AS1* on lung cancer risk.

## Materials and methods

### Study subjects

Our study was an ongoing hospital-based case control study. Quanto1.2.4 statistical software was used to calculate the sample size. Since we only found the incidence of lung cancer, we calculated the sample size according to the incidence of lung cancer. Lung cancer is divided into non-small cell lung cancer and small cell lung cancer. NSCLC includes four major types: adenocarcinoma, squamous cell carcinoma, large cell carcinoma, and adenosquamous carcinoma. We wanted to further explore the relationship between the risk of each type of pathology and rs2151280. So we performed pathological type analysis based on the collected patient information. We recruited 507 Han Chinese lung cancer patients and 542 Han Chinese healthy individuals in Shenyang City, located in northeast China. For excluding population admixture effects, we investigated the ethnicity of the participants’ parents for three generations. We perform a power calculation. The power was more than 0.88. All the cases were (1) newly diagnosed as lung cancer patients, (2) never accepted treatments, and (3) without cancer history or metastatic cancer. The healthy individuals were (1) selected in the same period in the same hospitals and (2) matched age and gender during the epidemiological survey. All enrolled subjects signed the informed consent forms and the institutional review board of China Medical University approved this study. Ten-milliliter venous blood sample collected from all enrolled subjects to detect the SNP. Phenol-chloroform method was used to isolate genomic DNA sample from venous blood. The rs2151280 was genotyped by 7500 Fast Real-time PCR system. In each genotyping process, blank controls were needed. The investigators randomly selected 10% samples to genotype twice for quality control, and the results were concordant with the duplicate samples.

### Statistical analysis

Student’s *t* test and χ2 test were calculated to analyze the difference in continuous and categorical variables between cases and healthy individuals, respectively. The goodness-of-fit χ2 test was used to examine the Hardy-Weinberg equilibrium (HWE) of rs2151280. We calculated the odds ratios (ORs) with their 95% confidence intervals (CIs) by unconditional logistic regression to evaluate the relationship between rs2151280 and lung cancer risk. The interaction of smoking status and rs2151280 on risk of lung cancer was evaluated by logistic regression on additive model and multiplicative model. The criterion of statistical significance was defined as *P* < 0.05, and all of the statistical tests were two-sided in this study. All the statistical analyses were calculated by SPSS software.

## Results

The demographic of 507 lung cancer cases and 542 healthy individuals was shown in Table 1. In terms of age, cases and controls seemed to be exactly matched (*P* = 0.096). There were no statistically significant differences in the proportion of sex status between the lung cancer case group (50.3% males and 49.7% females) and the control group (48.3% males and 51.7% females). As expected, the distribution of smoking status was significantly different in lung cancer cases and healthy controls (*P* < 0.001). Lung cancer cases included 248 adenocarcinomas, 125 squamous cell carcinomas, 90 small cell carcinomas, and 44 other types. The observed genotype frequencies for rs2151280 were in agreement with Hardy–Weinberg equilibrium. We wanted to further explore the relationship between the risk of each type of pathology and rs2151280. So, we performed pathological type analysis based on the collected patient information.

**Table 1:** Distribution of demographic variables in lung cancer and controls

Risk factor	Lung cancer	Controls	*P*
	(*N* = 507)	(*N* = 542)	
Age (mean ± SD)	59.20 ± 9.872	58.10 ± 11.504	0.096
Gender			
Male	255 (50.3%)	262 (48.3%)	0.527
Female	252 (49.7%)	280 (51.7%)	
Smoking status			
Ever	217 (42.8%)	66 (12.2%)	< 0.001
Never	290 (57.2%)	476 (87.8%)	
Stage			
I,II	93 (18.3%)		
III	146 (28.8%)		
IV	84 (16.6%)		
Missing	184 (36.3%)		
Pathological type			
AD	248 (48.92%)		
SQ	125 (24.65%)		
SCC	90 (17.75%)		
Else	44 (8.68%)		

*AD* lung adenocarcinoma, *SQ* lung squamous cell carcinoma, *SCC* small cell lung cancer

The association between rs2151280 and lung cancer risk was shown in Table 2. Compared with individuals carrying TT genotype, individuals with CC genotype of rs2151280 had a lower lung cancer risk (OR = 0.654, 95% CI = 0.443–0.965, *P* = 0.033). After adjusting for age, gender and smoking status risk factors, this association remained significant (OR = 0.640, 95% CI = 0.421–0.972, *P* = 0.036). In the recessive model, individuals carrying variant homozygote CC genotypes were associated with a significantly lower risk of lung cancer compared with individuals carrying heterozygote TC and TT genotype (adjusted OR = 0.648, 95%CI = 0.439–0.957, *P* = 0.029). In allele comparison, the C allele was associated with a lower risk of non-small-cell lung cancer (OR = 0.674, 95%CI = 0.560–0.812, *P* < 0.001) in Table 2. The associations of rs2151280 with lung adenocarcinoma (AD) and lung squamous cell carcinoma (SQ) were shown in Table 3. The statistically significant association was found in AD but not in SQ. Individuals with CC genotype had a 0.567-fold decreased risk of AD than those carrying TT genotype (95%CI = 0.333–0.965, *P* = 0.037). In the recessive model, individuals with CC genotype had a 0.543-fold decreased risk of AD than those carrying TT genotype or TC genotype (95%CI = 0.330–0.893, *P* = 0.016). However, there was no significant association between rs2151280 and the risk of SQ in the five models. We performed the stratification analyses to further estimate the association between rs2151280 and risk of lung cancer. In the subgroup analysis by gender, there was a significant correlation between rs2151280 and lung cancer risk in female (Table 4). Compared with TT genotype, variant CC genotype of 2151280 was associated with lower risk of lung cancer (adjusted OR = 0.533, 95 % CI = 0.310–0.916, *P* = 0.023). Compared with TT genotype and TC genotype, variant CC genotype was associated with lower risk of lung cancer and NSCLC (adjusted OR were 0.538 and 0.572, 95% CI were 0.326–0.888, and 0.335–0.975, *P* were 0.015 and 0.040, respectively).

**Table 2:** The association of the rs2151280 with lung cancer risk and non-small cell lung cancer

Genotyping		Lung cancer					Non-small-cell lung cancer				
	Controls (%)	Cases (%)	OR (95%CI)	*P* value	ORª (95%CI)	*P*ª value	Cases (%)	OR (95%CI)	*P* value	ORª (95%CI)	*P*ª value
TT	203 (37.5)	207 (40.8)	1.00 (ref)				153 (38.9)	1.00 (ref)			
TC	255 (47.0)	244 (48.1)	0.938 (0.723–1.219)	0.633	0.976 (0.737–1.292)	0.865	196 (49.9)	1.020 (0.770–1.350)	0.891	1.072 (0.791–1.453)	0.654
CC	84 (15.5)	56 (11.0)	0.654 (0.443-0.965)	0.033	0.640 (0.421–0.972)	0.036	44 (11.2)	0.695 (0.456–1.059)	0.090	0.698 (0.444–1.098)	0.120
CC+TC vs TT			0.868 (0.677–1.112)	0.263	0.890 (0.682–1.162)	0.392		0.939 (0.719–1.227)	0.646	0.976 (0.731–1.304)	0.871
CC vs TC+TT			0.677 (0.471–0.973)	0.035	0.648 (0.439–0.957)	0.029		0.687 (0.465–1.016)	0.060	0.672 (0.441–1.022)	0.063
T allele	661 (61.0)	658 (64.9)	1.00 (ref)				502 (63.9)	1.00 (ref)			
C allele	423 (39.0)	356 (35.1)	0.845 (0.708–1.010)	0.064			284 (36.1)	0.674 (0.560–0.812)	0.001		

*OR* odds ratio, *CI* confidence interval

^a^Adjusted for age, gender, and smoking

**Table 3:** The association of the rs2151280 with lung adenocarcinoma risk and lung squamous cell carcinoma risk

Genotyping		Lung adenocarcinoma					Lung squamous cell carcinoma				
	Controls (%)	Cases (%)	OR (95%CI)	*P* value	ORª (95%CI)	*P*ª value	Cases (%)	OR (95%CI)	*P* value	ORª (95%CI)	*P*ª value
TT	203 (37.5)	98 (39.5)	1.00 (ref)					1.00 (ref)			
TC	255 (47.0)	126 (50.8)	1.024 (0.742–1.412)	0.887	1.082 (0.773–1.513)	0.646	43 (34.4)	1.203 (0.785–1.845)	0.396	1.530 (0.893–2.621)	0.122
CC	84 (15.5)	24 (9.7)	0.592 (0.354–0.989)	0.045	0.567 (0.333–0.965)	0.037	65 (52.0)	0.955 (0.516–1.770)	0.885	1.343 (0.615–2.932)	0.459
CC+TC vs TT			0.917 (0.673–1.248)	0.580	0.948 (0.688–1.306)	0.743	17 (13.6)	1.142 (0.759–1.717)	0.524	1.487 (0.889–2.488)	0.130
CC vs TC+TT			0.584 (0.361–0.945)	0.028	0.543 (0.330–0.893)	0.016		0.858 (0.489–1.505)	0.594	1.053 (0.518–2.143)	0.886
T allele	661 (61.0)	322 (64.9)	1.00 (ref)				151 (60.4)	1.00 (ref)			
C allele	423 (39.0)	174 (35.1)	0.844 (0.677–1.053)	0.134			99 (39.6)	1.025 (0.773–1.357)	0.866		

*OR* odds ratio, *CI* confidence interval

^a^Adjusted for age, gender, and smoking

**Table 4:** The association of the rs2151280 with lung cancer risks and NSCLC in female and male population

Genotyping		Lung cancer					Non-small-cell lung cancer				
	Controls (%)	Cases (%)	OR (95%CI)	*P* value	ORª (95%CI)	*P*ª value	Cases (%)	OR (95%CI)	*P* value	ORª (95%CI)	*P*ª value
Female											
TT	104 (37.1)	102 (40.5)	1.00 (ref)				73 (37.8)	1.00 (ref)			
TC	123 (43.9)	122 (48.4)	1.011 (0.698–1.465)	0.953	0.984 (0.676–1.434)	0.935	97 (50.3)	1.124 (0.753–1.677)	0.569	1.109 (0.740–1.661)	0.616
CC	53 (18.9)	28 (11.1)	0.539 (0.316–0.918)	0.023	0.533 (0.310–0.916)	0.023	23 (11.9)	0.618 (0.348–1.097)	0.100	0.606 (0.339–1.081)	0.090
CC+TC vs TT			0.869 (0.613–1.232)	0.431	0.850 (0.596–1.211)	0.368		0.971 (0.665–1.419)	0.880	0.957 (0.653–1.402)	0.822
CC vs TC+TT			0.535 (0.327–0.877)	0.013	0.538 (0.326–0.888)	0.015		0.579 (0.342–0.983)	0.043	0.572 (0.335–0.975)	0.040
T allele	331 (59.1)	326 (64.7)	1.00 (ref)				243 (63.0)	1.00 (ref)			
C allele	229 (40.9)	178 (35.3)	0.789 (0.616–1.012)	0.062			143 (37.0)	0.851 (0.652–1.110)	0.234		
Male											
TT	99 (37.8)	105 (41.2)	1.00 (ref)					1.00 (ref)			
TC	132 (50.4)	122 (47.8)	0.871 (0.603–1.260)	0.464	0.929 (0.570–1.515)	0.769	80 (40.0)	0.928 (0.626–1.375)	0.710	1.078 (0.624–1.862)	0.788
CC	31 (11.8)	28 (11.0)	0.852 (0.477–1.521)	0.587	0.724 (0.336–1.558)	0.408	99 (49.5)	0.838 (0.448–1.570)	0.582	0.743 (0.315–1.752)	0.497
CC+TC vs TT			0.868 (0.610–1.235)	0.431	0.886 (0.555–1.4130)	0.611	21 (10.5)	0.911 (0.625–1.329)	0.628	1.004 (0.596–1.692)	0.989
CC vs TC+TT			0.919 (0.534–1.582)	0.761	0.753 (0.367–1.545)	0.440		0.874 (0.486–1.573)	0.654	0.713 (0.320–1.590)	0.408
T allele	330 (63.0)	332 (65.1)	1.00 (ref)				259 (64.8)	1.00 (ref)			
C allele	194 (37.0)	178 (34.9)	0.912 (0.707–1.176)	0.477			141 (35.2)	0.926 (0.706–1.214)	0.579		

*OR* odds ratio, *CI* confidence interval, *NSCLC* non-small-cell lung cancer

^a^Adjusted for age, gender, and smoking

However, in the subgroup of age, we failed to find any statistically significant associations between rs2151280 and the risk of lung cancer and NSCLC (Table 5).

**Table 5:** Stratified analyses of the rs2151280 with lung cancer risk and NSCLC by age

Genotyping		Lung cancer					Non-small-cell lung cancer				
	Controls (%)	Cases (%)	OR (95%CI)	*P* value	ORª (95%CI)	*P*ª value	Cases (%)	OR (95%CI)	*P* value	ORª (95%CI)	*P*ª value
> 60											
TT	95 (36.8)	106 (40.9)	1.00 (ref)				83 (38.1)	1.00 (ref)			
TC	117 (45.3)	123 (47.5)	0.942 (0.647–1.371)	0.756	0.922 (0.617–1.380)	0.695	110 (50.5)	1.076 (0.726–1.594)	0.715	1.054 (0.690–1.611)	0.807
CC	46 (17.8)	30 (11.6)	0.584 (0.324–1.000)	0.050	0.622 (0.350–1.108)	0.107	25 (11.5)	0.622 (0.352–1.099)	0.102	0.660 (0.358–1.218)	0.184
CC+TC vs TT			0.841 (0.590–1.199)	0.339	0.840 (0.574–1.229)	0.369		0.948 (0.653–1.376)	0.779	0.946 (0.633–1.414)	0.786
CC vs TC+TT			0.604 (0.368-0.992)	0.046	0.65 (0.381–1.109)	0.114		0.597 (0.353–1.009)	0.054	0.641 (0.365–1.128)	0.123
T allele	307 (59.5)	335 (64.7)	1.00 (ref)				276 (63.3)	1.00 (ref)			
C allele	209 (40.5)	183 (35.3)	0.802 (0.624–1.032)	0.086			160 (36.7)	0.852 (0.655–1.107)	0.230		
≤ 60											
TT	108 (38.0)	101 (40.7)	1.00 (ref)				70 (40.0)	1.00 (ref)			
TC	138 (48.6)	121 (48.8)	0.938 (0.651–1.351)	0.729	1.079 (0.717–1.622)	0.716	86 (49.1)	0.961 (0.642–1.440)	0.849	1.151 (0.730–1.815)	0.544
CC	38 (13.4)	26 (10.5)	0.732 (0.415–1.291)	0.281	0.660 (0.353–1.234)	0.193	19 (10.9)	0.771 (0.412–1.445)	0.418	0.763 (0.383–1.521)	0.443
CC+TC vs TT			0.893 (0.630–1.266)	0.525	0.976 (0.662–1.439)	0.904		0.920 (0.626–1.354)	0.674	1.056 (0.685–1.628)	0.805
CC vs TC+TT			0.758 (0.446–1.289)	0.307	0.633 (0.353–1.136)	0.125		0.788 (0.439–1.417)	0.427	0.707 (0.372–1.342)	0.288
T allele	354 (62.3)	323 (65.1)	1.00 (ref)				226 (64.6)	1.00 (ref)			
C allele	214 (37.7)	173 (34.9)	0.886 (0.689–1.139)	0.344			124 (35.4)	0.908 (0.688–1.197)	0.493		

*OR* odds ratio, *CI* confidence interval, *NSCLC* non-small-cell lung cancer

^a^Adjusted for age, gender, and smoking

In the subgroup of smoking, we found the significant association among rs2151280 with the risk of lung cancer and NSCLC in the individuals who had never exposed to smoking (Table 6). Carriers of the rs2151280 CC genotype had a lower lung cancer risk than carriers with TT genotype (adjusted OR = 0.518, 95%CI = 0.316–0.851, *P* = 0.009). Individuals carrying variant homozygote CC genotype had decreased risk of lung cancer by 0.561-fold (adjusted OR = 0.561, 95%CI = 0.354–0.891, *P* = 0.014) compared individuals carrying with heterozygote TC and TT genotype. *CDKN2B-AS1* rs2151280 was significantly associated with the risk of NSCLC (CC vs TT: OR = 0.572, 95%CI = 0.334–0.980, *P* = 0.042; CCvsTC+TT: OR = 0.587, 95%CI = 0.356–0.968, *P* = 0.037, respectively). Tables 7, 8, 9 had shown the interaction between rs2151280 and smoking exposure on the susceptibility of lung cancer, NSCLC, AD, and SQ on additive interaction. Compared to individuals with CC genotype and never exposure to smoking, individuals with TC/TT genotype and never smoking had the increased risk of lung cancer and lung adenocarcinoma (adjusted OR = 1.659, 95%CI = 1.056–2.606, *P* = 0.028; OR = 1.814, 95%CI = 1.042–3.156, *P* = 0.035, respectively). Compared to individuals with CC genotype and never exposure to smoking, individuals with TC/TT genotype and ever smoking had the increased risk of lung cancer and NSCLC. At the same time, the same results existed in AD and SQ (adjusted OR = 7.403, 95%CI = 3.815–14.366, OR = 12.139, 95%CI = 4.827–30.526, respectively). However, the results of quantitative analyses indicated that there were no significant (Table 9). There were three measures [relative excess risk due to interaction (RERI), attributable proportion due to interaction (AP), and synergy index (S)] with their 95% CI were used to show the relationship. The criterion of these three measures was just as our previous study [28]. Due to the *P* value was more than 0.05, the results of multiplicative interaction indicated that there were no association between rs2151280 risk genotypes with smoking exposure and lung cancer risk.

**Table 6:** Stratified analyses of the rs2151280 with lung cancer risks and NSCLC by smoking

Genotyping		Lung cancer					Non-small-cell lung cancer				
	Controls (%)	Cases (%)	OR (95%CI)	*P* value	ORª (95%CI)	*P*ª value	Cases (%)	OR (95%CI)	*P* value	ORª (95%CI)	*P*ª value
Never											
TT	172 (36.1)	121 (41.7)	1.00 (ref)				87 (39.7)	1.00 (ref)			
TC	229 (48.1)	138 (47.6)	0.857 (0.626–1.173)	0.334	0.865 (0.624–1.201)	0.387	107 (48.9)	0.924 (0.654–1.305)	0.653	0.954 (0.665–1.368)	0.798
CC	75 (15.8)	31 (10.7)	0.588 (0.364–0.948)	0.029	0.518 (0.316–0.851)	0.009	25 (11.4)	0.659 (0.391–1.109)	0.117	0.572 (0.334–0.980)	0.042
CC+TC vs TT			0.790 (0.586–1.066)	0.123	0.773 (0.567–1.056)	0.106		0.858 (0.618–1.193)	0.363	0.850 (0.604–1.197)	0.351
CC vs TC+TT			0.640 (0.409–1.000)	0.050	0.561 (0.354–0.891)	0.014		0.689 (0.425–1.118)	0.131	0.587 (0.356–0.968)	0.037
T allele	573 (60.2)	380 (65.5)	1.00 (ref)				281 (64.2)	1.00 (ref)			
C allele	379 (39.8)	200 (34.5)	0.796 (0.642–0.986)	0.037			157 (35.8)	0.845 (0.668–1.068)	0.158		
ever											
TT	31 (47.0)	86 (39.6)	1.00 (ref)				66 (37.9)	1.00 (ref)			
TC	26 (39.4)	106 (48.8)	1.470 (0.812–2.661)	0.204	1.477 (0.762–2.862)	0.248	89 (51.1)	1.608 (0.873–2.961)	0.128	1.812 (0.894–3.673)	0.099
CC	9 (13.6)	25 (11.5)	1.001 (0.421–2.379)	0.998	0.992 (0.378–2.608)	0.987	19 (10.9)	0.992 (0.403–2.440)	0.985	1.025 (0.366–2.872)	0.962
CC+TC vs TT			1.349 (0.775–2.349)	0.290	1.351 (0.728–2.508)	0.340		1.449 (0.818–2.569)	0.204	1.599 (0.826–3.094)	0.164
CC vs TC+TT			0.825 (0.364–1.867)	0.644	0.815 (0.328–2.027)	0.660		0.776 (0.332–1.815)	0.559	0.756 (0.287–1.987)	0.570
T allele	88 (66.7)	278 (64.1)	1.00 (ref)				221 (63.5)	1.00 (ref)			
C allele	44 (33.3)	156 (35.9)	1.122 (0.744–1.694)	0.583			127 (36.5)	1.149 (0.753–1.754)	0.518		

*OR* odds ratio, *CI* confidence interval, *NSCLC* non-small-cell lung cancer

^a^Adjusted for age, gender, and smoking

**Table 7:** Relationship of interaction between rs2151280 and smoking with lung cancer risk and NSCLC

	Controls (%)	Smoking		Lung Cancer					Non-small cell lung cancer			
			Cases (%)	OR (95%CI)	*P* value	ORª (95%CI)	*P*^a^ value	Cases (%)	OR (95%CI)	*P* value	ORª (95%CI)	*P*^a^ value
CC	75 (13.8)	Never	31 (6.1)	1.00 (ref)				25 (6.4)	1.00 (ref)			
TC+TT	401 (74.0)	Never	259 (51.1)	1.563 (1.000–2.443)	0.050	1.659 (1.056–2.606)	0.028	194 (49.4)	1.451 (0.894–2.355)	0.131	1.559 (0.956–2.542)	0.075
CC	9 (1.7)	Ever	25 (4.9)	6.72 (2.818–16.029)	< 0.001	9.392 (3.849–22.914)	< 0.001	19 (4.8)	6.333 (2.541–15.784)	< 0.001	8.42 (3.296–21.510)	< 0.001
TC+TT	57 (10.5)	Ever	192 (37.9)	8.149 (4.882–13.604)	< 0.001	11.442 (6.626–19.761)	< 0.001	155 (39.4)	8.158 (4.73–14.07)	< 0.001	10.905 (6.100–19.495)	< 0.001

*OR* odds ratio, *CI* confidence interval, *NSCLC* non-small-cell lung cancer

^a^Adjusted for age, gender, and smoking

**Table 8:** Relationship of interaction between rs2151280 and smoking with lung adenocarcinoma risk and lung squamous cell carcinoma risk

	Controls (%)	Smoking	Lung adenocarcinoma					Lung squamous cell carcinoma				
			Cases (%)	OR (95%CI)	*P* value	ORª (95%CI)	*P*^a^ value	Cases (%)	OR (95%CI)	*P* value	ORª (95%CI)	*P*^a^ value
CC	75 (13.8)	Never	18 (7.3)	1.00 (ref)				6 (4.8)	1.00 (ref)			
TC+TT	401 (74.0)	Never	158 (63.7)	1.642 (0.951–2.835)	0.075	1.814 (1.042–3.156)	0.035	27 (21.6)	0.842 (0.336–2.108)	0.713	0.717 (0.280–1.831)	0.486
CC	9 (1.7)	Ever	6 (2.4)	2.778 (0.876–8.808)	0.083	3.763 (1.149–12.323)	0.029	11 (8.8)	15.278(4.549–51.306)	< 0.001	9.537 (2.716–33.490)	< 0.001
TC+TT	57 (10.5)	Ever	66 (26.6)	4.825 (2.583–9.011)	0.000	7.403 (3.815–14.366)	< 0.001	81 (64.8)	17.763 (7.237–43.600)	< 0.001	12.139 (4.827–30.526)	< 0.001

*OR* odds ratio, *CI* confidence interval

^a^Adjusted for age, gender, and smoking

**Table 9:** Interaction measures between rs2151280 in lncRNA and smoking exposure on lung cancer, NSCLC, lung adenocarcinoma, and lung squamous cell carcinoma

	Lung cancer			NSCLC			Lung adenocarcinoma		Lung squamous cell carcinoma		
Measure	Estimate	95%CI	Measure	Estimate	95%CI	Measure	Estimate	95%CI	Measure	Estimate	95%CI
RERI	0.866	− 4.798–6.531	RERI	1.373	− 4.235–6.982	RERI	1.405	− 1.955− 4.765	RERI	2.644	− 12.154− 17.441
AP	0.106	− 0.578–0.791	AP	0.168	− 0.500–0.837	AP	0.291	− 0.366–0.949	AP	0.149	− 0.663–0.960
S	1.138	0.469–2.760	S	1.237	0.483–3.169	S	1.581	0.428–5.837	S	1.187	0.428–3.293

*NSCLC* non-small cell lung cancer, *RERI* relative excess risk due to interaction, *AP* attributable proportion due to interaction, *S* synergy index, *95%CI* 95% confidence interval

## Discussion

Lung cancer is one of the most common malignancies, with higher morbidity and higher mortality in the world in the 21st century [29, 30]. According to reports, the crude incidence of lung cancer in developing countries is increasing rapidly. Data was recorded by the International Agency for Research on Cancer (IARC) from 2008 to 2012. The data had shown that lung cancer was the first common malignancies in Chinese male (52.7 cases per 100,000 men) and the second common malignancies in Chinese female (28.4 cases per 100,000 women is just less than breast cancer). The age-standardized incidence rate (ASR) world was 45.9 per 100,000 s and 38.6 per 100,000 s for men in the USA and UK, respectively. Due to the limitations of cancer detection, lung cancer was often too late to get effective treatment. LncRNAs, one of the most important classes of ncRNAs with a length of more than 200 bp, became a hotpot as potentially critical regulators in cellular processes. The association between lncRNA *DGCR5* and lung cancer had been found [31]. Many studies had shown that lncRNAs are associated with lung cancer, such as *H19* [32], *ATB* [33], *CDKN2B-AS1* [22], and *MALAT1* [34].

*CDKN2B-AS1* is located within the *CDKN2B-CDKN2A* gene cluster at chromosome 9p21. *CDKN2B-AS1* was transcribed and sewn into a complex pattern [35] and play a role in a variety of cell functions [36]. Compared with healthy individuals, the high expression of lncRNA *CDKN2B-AS1* was significantly existed in NSCLC patients [19]. In addition, the area under the curve (AUC) of *CDKN2B-AS1* was greater than tumor markers (*CEA*, *CYFRA21-1*, and *SCCA*). It was indicating that *CDKN2B-AS1* had a significant efficacy in distinguishing NSCLC patients and controls [19]. AUC had widely recognized as an inherent accuracy index for the authenticity evaluation of diagnostic tests. When the value of AUC is 0.5, there is no diagnostic value. When the value of AUC is 1, the diagnostic value is high. A previous study by Nie.et al had reported that *CDKN2B-AS1* could promote the proliferation of non-small cell lung cancer cells and inhibit cell apoptosis [17].

In this study, we demonstrated that the rs2151280 in lncRNA *CDKN2B-AS1* was significantly associated with lung cancer. The minor allele frequency (MAF) of rs2151280 was 0.268 in Chinese population. This study was the first time to suggest that rs2151280 might be an important risk factor in lung cancer. Specifically, the results of our study suggested that rs2151280 CC genotype could be a protecting factor in lung cancer compared with TT genotype, especially among adenocarcinoma patients. Compared with TC/TT genotypes, CC genotype had a decreased risk of lung cancer including adenocarcinoma patients. The C allele had reduced the risk of NSCLC. Furthermore, in the subgroup of gender, individuals with CC genotype had a lower risk of lung cancer compared with individuals with TT genotype (adjusted *P* = 0.023) in female. In female, individuals with CC genotype also had a lower risk of lung cancer and NSCLC compared with individuals with /TCTT genotypes (adjusted *P* = 0.015 or *P* = 0.040, respectively). Moreover, in the subgroup of smoking status, the same results were existed in lung cancer in never smoking population. However, we failed to find the any statistically significant association between rs2151280 and SQ. In this study, the sample size might be too small to get the significant results in SQ. The interaction of gene–environment on the risk of cancer was studied by few studies. Therefore, in this case-control study, we investigated the interaction between smoking status exposure and the rs2151280 C/T on lung cancer risk. The crossover analysis suggested that the interaction between rs2151280 and smoking exposure on the susceptibility of lung cancer, NSCLC, AD, and SQ were existed in Chinese population. However, the quantitative analyses had shown that no any statistical significance existed on the interaction between the rs2151280 and smoking status exposure.

Admittedly, this present study had several limitations. Firstly, the cases and controls were selected from hospitals, which might lead to Berkson’s bias. As such, the cases and controls selected from several different hospitals to decrease this bias. Secondly, smoking exposure data were collected by interviewing participants, which easily lead to recall bias. Thirdly, the sample size was small in the subgroup analysis and interaction analysis of this study, and the research results had certain limitations. Finally, due to the small number of genes in this study, polygenic score was not studied. In future studies, we will consider calculating the polygenic score to make our study results more meaningful. Therefore, the relationship between the rs2151280 in lncRNA *CDKN2B-AS1* and lung cancer risk need to be validated by further large size studies.

## Conclusions

Our findings provided new insights into the roles of rs2151280 in lung cancer risk in northern Chinese Han population. This hospital-based case-control study suggested that *CDKN2B-AS1* rs2151280 T>C was associated with the risk of lung cancer. However, the gene–environment interaction between rs2151280 and smoking exposure was not statistically significant in this study.

## Data Availability

Not applicable.

## Abbreviations

AD: Lung adenocarcinoma
ASR: Age-standardized incidence rate
BCC: Basal cell carcinoma
CIs: Confidence intervals
ESCC: Esophageal squamous cell carcinoma
GWASs: Genome-wide association studies
HWE: Hardy-Weinberg equilibrium
IARC: International Agency for Research on Cancer
lncRNAs: Long non-coding RNAs
MAF: Minor allele frequency
NSCLC: Non-small cell lung cancer
ORs: Odds ratios
PBMCs: Peripheral blood mononuclear cells
PNF: Plexiform neurofibromas
SQ: Lung squamous cell carcinoma
